# Frictions with Ice in Tic Douleureux

**Published:** 1872-10

**Authors:** 


Frictions with Ice in Tic Douleureux.—Dr. Winternitz, of Vienna (*Berliner Klinische Wochenschrift*, No. 33, 1872), in a late meeting of the Association of Physicians of that city, relates that he saw an unusually stubborn and severe case of tic douleureux at last cured by rubbing the diseased half of the face with smooth pieces of ice. This method, first used by Dr. Diakowski, consists in rubbing the diseased side of the face in regular, uniform motions, with a smooth piece of ice, for five or six minutes—at first in intervals of the same length of time, which are gradually increased as the process of rubbing is continued. In this case, the pain disappeared after twelve hours, and has not returned since then, a period of ten months. During the frictions, the patient holds an alcoholic fluid in the cavity of the mouth, whereby the painfulness of the manipulation is considerably reduced. R.

